# New Jersey State Dental Society

**Published:** 1871-03

**Authors:** E. F. Hanks


### Proceedings of Societies.

### NEW JERSEY STATE DENTAL SOCIETY.

Pursuant to a call signed by twenty-seven dentists of the State, the Convention met at Trenton, and formed the *State Dental Society*, October 25th, 1870.

Dr. Fowler was called to the chair, and Dr. Hanks selected as Secretary.

A committee was then appointed, to report a Constitution and By-laws for the use of the Society, viz: Drs. A. W. Kingsly, J. Hayhurst and C. S. Stockton. who subsequently reported a Preamble and Constitution, which declares among the objects of this Society, to cultivate the science and art of dentistry, and all collateral branches, and to elevate and sustain the professional character of dentists, and promote amongst them mutual improvement, social intercourse, and good will.

The officers are a President, Vice-President, Secretary and Treasurer.

Three classes of membership are provided for: active, corresponding and honorary.

Active members must be graduates of some dental college, or have been in practice three years, including term of pupilage, subject to examination by the Committee on Membership

The Constitution and By-laws were adopted, and an election of officers ordered with the following result:

*President*—J. Hayhurst, Lambertville.

*Vice-President*—C. S. Stockton, Mt. Holly.

*Secretary*—E. F. Hanks, Rahway.

*Treasurer*—L. E. Reading, Trenton.

Dr. Kingsly, moved that a vote of thanks be tendered Dr. Hanks for his services in bringing us together for so laudable a purpose.

Dr. Kingsly moved that Dr. Stockton conduct the President to the chair, which he assumed with a few remarks thanking the gentlemen for the honor of presiding over their first meeting.

Professors J. Taft, of Cincinnati, and J. H. McQuillen, of Philadelphia, were proposed as honorary members, by Drs. Hanks and Stockton, respectively; also, Dr. J. S. Latimer, of New York city, as corresponding member, by Dr. Cosad.

They were all unanimously elected.

SECOND DAY.

President in the chair.

Minutes read and approved.

The Society proceeded to business, by selecting Newark as the next place of meeting, second Tuesday, July, 1871.

Dr. Reading was authorized to present a notice of the meeting to the Trenton papers.

Dr. Stockton was selected to deliver the annual address at the next meeting.

Operative dentistry being the first in order, the application of the rubber dam, was announced as the first subject for discussion.

Dr. Kingsly, by request, gave a clinic, demonstrating its usefulness, remarking, at the same time, that if he were compelled to give up all he had learned in the last ten years, with the privilege of retaining one thing, that would be the rubber dam.

The next subject was the advantages of the different methods of separating teeth.

Dr. Stockton separates the bicuspids of children, after the method of Dr. Author, of Baltimore, and advised it in all cases of frail or very soft teeth.

Dr. Hanks objected to the V shaped separations, on account of the packing of food between the teeth, causing inflammation.

Dr. Kingsley, advocated a mixed practice, according to the demand of the case, relying on his judgment.

Dr. DeLange separates incisors by packing cotton between them.

Dr. Hanks advocated quick wedging, using generally two wedges, one at the points, and one at the necks.

Dr. Stockton objected from personal experience, on account of the severe pain.

Dr. Cosad thought the quick method preferable when judiciously applied, but when carried to the extent of throwing all the spaces into one, he agreed with Dr. Frank Abbot, that the practice was villainous. After a few moments the pain subsides, leaving a numbness that is not unpleasant.

Dr. Dibble separates bicuspids by the use of the inverted V shape.

Dr. Hayhurst separates with orangewood, applied by the patient. Objected to filing incisors, on account of the unsightliness of their appearance.

Mechanical dentistry was next taken up.

Dr. Dean took the floor, and explained the method of taking plaster impressions used by Dr. Wescott, of Syracuse.

Dr. Chen fully indorsed the same.

Dr. Stockton, in very soft and flabby mouths, trims the cast in such manner as to allow the plate to rest firmly against the palatine surface.

On motion, adjourned to meet at Newark, second Tuesday, July, 1871.

E. F. Hanks, Sec.

